# Assembly and comparative analysis of the first complete mitochondrial genome of zicaitai (*Brassica rapa* var. *Purpuraria*): insights into its genetic architecture and evolutionary relationships

**DOI:** 10.3389/fpls.2024.1475064

**Published:** 2024-10-10

**Authors:** Wanyu Xiao, Xian Wu, Xianyu Zhou, Jing Zhang, Jianghua Huang, Xiuchun Dai, Hailong Ren, Donglin Xu

**Affiliations:** ^1^ Guangzhou Municipal Crop Seed Quality Inspection Center, Guangzhou Academy of Agricultural and Rural Sciences, Guangzhou, China; ^2^ Northeast Agricultural University, Harbin, China; ^3^ College of Agriculture and Biology, Zhongkai University of Agriculture and Engineering, Guangzhou, China; ^4^ Guangdong Provincial Key Laboratory of Crop Genetic Improvement, Crops Research Institute, Guangdong Academy of Agricultural Sciences, Guangzhou, China

**Keywords:** zicaitai, *Brassica*, mitochondrial genome, phylogenetic analysis, gene transfer

## Abstract

**Introduction:**

Zicaitai (*Brassica rapa* var. *purpuraria*) is a *Brassica* variety renowned for its distinctive taste and rich nutritional profile. In recent years, the mitochondrial genomes of several *Brassica* species have been documented, but the mitogenome of Zicaitai remains unreported.

**Methods:**

In this study, we characterized the Zicaitai mitogenome achieved through the assembly of sequencing reads derived from both the Oxford Nanopore and Illumina platforms. A detailed comparative analysis was carried out with other *Brassica* species to draw comparisons and contrasts. In-depth analyses of codon usage patterns, instances of RNA editing, and the prevalence of sequence repeats within the mitogenome were also conducted to gain a more nuanced understanding of its genetic architecture. A phylogenetic analysis was performed, utilizing the coding sequences (CDS) from the mitochondrial genome of Zicaitai and that of 20 closely related species/varieties to trace evolutionary connections.

**Results:**

The Zicaitai mitogenome is characterized by a circular structure spanning 219,779 base pairs, and it encompasses a total of 59 genes. This gene set includes 33 protein-coding genes, 23 tRNA genes, and 3 rRNA genes, providing a rich foundation for further genomic study. An analysis of the Ka/Ks ratios for 30 protein-coding genes shared by the mitogenomes of Zicaitai and seven other *Brassica* species revealed that most of these genes had undergone purifying selection. Additionally, the study explored the migration of genes between the chloroplast and nuclear genomes and the mitogenome, offering insights into the dynamics of genetic exchange within the *Brassica* genus.

**Discussion:**

The collective results in this study will serve as a foundational resource, aiding future evolutionary studies focused on *B. rapa*, and contributing to a broader understanding of the complexities of plant evolution.

## 1 Introduction

*Brassica* is a critically important genus within the *Brassicaceae* family (Essoh et al., 2020). It encompasses six principal species: *Brassica rapa* (AA, 2n = 2x = 20), *B. oleracea* (CC, 2n = 2x = 18), *B. nigra* (BB, 2n = 2x = 16), *B. napus* (AACC, 2n = 4x = 38), *B. juncea* (AABB, 2n = 4x = 36) and *B. carinata* (BBCC, 2n = 4x = 34) (Koh et al., 2017; Zhang et al., 2022). The genetic interrelations among these species are often depicted as the ‘Triangle of U’ (Wu et al., 2022). Zicaitai (*B. rapa* var. *purpuraria*), a variety of *B. rapa* that originated in Central China as a result of extensive cultivation and domestication (Li et al., 2019), is widely cultivated in southern China. This variety is a popular vegetable nationwide, celebrated for its distinctive flavor profile and substantial nutritional benefits, particularly because of its rich anthocyanin content in the leaves and stalks (Podsędek, 2017; Guo et al., 2015). Anthocyanins, known for their diverse biological roles in plants and their health benefits to humans (Hayashi et al., 2010; Lee et al., 2017; Zhang and Jing, 2022; Nistor et al., 2022), have propelled zicaitai into the spotlight in recent years (Guo et al., 2015; Li et al., 2019; Zhang X. et al., 2020).

Mitochondria are semi-autonomous organelles found in the majority of eukaryotic cells (Ye et al., 2017) and perform a range of essential functions, including energy conversion, tricarboxylic acid cycling, oxidative phosphorylation, calcium ion storage, cell proliferation and metabolism, etc (Hamani and Giege, 2014; Møller et al., 2021). Plant mitochondrial genomes are considerably variable in length, gene arrangement, and gene content (Birky, 1995; Bi et al., 2020), and they contain large repeats that can result in isomerization within a species. Recent studies have also highlighted the frequent gene transfers between nuclear, mitochondrial, and chloroplast genomes as a common phenomenon during plant evolution (Chang et al., 2013; Zhao et al., 2019). Given the substantial impact of plant organellar genomes on metabolism and adaptation, a detailed analysis of their sequences and structures can shed light on the evolutionary history and the intricate interplay between these genomes (Pinard et al., 2019).

Leveraging the advancements in sequencing technology, numerous *Brassica* mitochondrial genomes have been characterized, including the mitogenomes of several subspecies in *B. rapa* (Handa, 2003; Chang et al., 2011; Chen et al., 2011; Wu et al., 2019; Shao et al., 2021). However, the situation among the subspecies/varieties of *B. rapa* remains confusing, especially for the Zicaitai, whose selection and domestication are still unknown. Studying the mitochondrial genome of Zicaitai is of significant value for understanding its evolutionary position within the species, genetic variation, phylogeny, and domestication history of the Chinese cabbage varieties. However, the mitochondrial genome of zicaitai remains largely uncharted territory. In this study, we sequenced and annotated the its mitogenome, conducting a detailed analysis of its genomic features, codon usage, RNA editing, sequence repeats, and comparative genomics. The findings provide an in-depth understanding of the zicaitai mitochondrial genome, which is expected to bolster ongoing research efforts into the evolution of *B. rapa*.

## 2 Materials and methods

### 2.1 Plant materials and DNA sequencing

Zicaitai seeds, designated ‘Youxuan hongshan’ were sourced from the Guangzhou Academy of Agricultural Sciences. Germination of the seeds was initiated at 25°C within a growth chamber, followed by transplantation into a greenhouse under a 16/8-hour light-dark photoperiod at a temperature of 25°C, for a 6-week growth. Twenty grams of zicaitai leaf tissue from one single well-grown seedling was then harvested, promptly transported using dry ice, and dispatched to Genepioneer Biotechnologies Company in Nanjing, China. The integrity of the DNA samples was assessed via agarose gel electrophoresis, and their concentrations were quantified using a Nanodrop instrument (model 2000c UV-Vis). Samples meeting the quality criteria were subsequently submitted for sequencing using both Oxford Nanopore and Illumina platforms.

### 2.2 Mitogenome assembly and annotation

Given the high conservation of plant mitochondrial genes, including coding sequences (CDS) and ribosomal RNA (rRNA), the minimap2 software (version 2.1) (Li, 2018), a tool designed for tri-generational comparison, was utilized. This software compared the original tri-generational data against a reference gene sequence, with preference given to sequences exceeding 50 base pairs in length as candidate sequences. The seed sequence was chosen based on its enrichment in core genes and superior relative quality, indicating a more complete representation of the core genes. Subsequently, minimap2 was employed to align the original sequencing data with the seed sequence, and sequences with an overlap exceeding 1 kilobase and a similarity higher than 70% were integrated into the seed sequence. This iterative comparison of the original data with the seed sequence facilitated the acquisition of comprehensive mitochondrial genome data. The tri-generational assembly software Canu (Koren et al., 2017) was then applied to refine the obtained data. Bowtie2 (version 2.3.5.1) (Langmead and Salzberg, 2012) was used to align the second-generation data with the corrected sequence, followed by the application of Unicycler (version 0.4.8) (Wick et al., 2017) to align the second-generation data with the corrected tri-generational data. The final assembly was manually curated to determine the orientation of the branches. The mitochondrial genome map was generated using OGDRAW (https://chlorobox.mpimp-golm.mpg.de/OGDraw.html) (Lohse et al., 2007). Mitochondrial annotation proceeded through the following steps: (1) BLAST was employed to align published plant mitochondrial sequences with their respective encoded proteins and rRNA, with subsequent manual refinements based on related species; (2) Transfer RNA (tRNA) was annotated using tRNAscanSE (http://lowelab.ucsc.edu/tRNAscan-SE/) (Chan and Lowe, 2019); (3) Open Reading Frames (ORFs) were annotated with the Open Reading Frame Finder (http://www.ncbi.nlm.nih.gov/gorf/gorf.html), considering the shortest length of 102 base pairs. For the purpose of collinearity analysis, the nucmer (4.0.0beta2) software was executed utilizing the “–maxmatch” parameter to facilitate genomic alignments between the assembled mitochondrial sequence of zicaitai and those of related species or varieties, resulting in the creation of Dot-plot graphs.

### 2.3 Analysis of repeat structures and SSRs

Dispersed repeats were identified using the REPuter tool (Kurtz et al., 2001); tandem repeats were detected with the Tandem Repeats Finder software (trf409.linux64: 2 7 7 80 10 50 2000 -f -d -m) (Benson, 1999); and simple sequence repeats (SSRs) were analyzed using the MISA program (Beier et al., 2017), with a minimum distance between SSRs set at 100 base pairs and the motif size for one- to six-nucleotide SSRs set to 10, 5, 4, 3, 3, and 3, respectively.

### 2.4 Prediction of RNA editing sites and codon usage

The codon composition within the mitochondrial genome of zicaitai was examined using a custom Perl script, which was designed to identify unique coding sequences (CDS), determine the number of codons per gene, and calculate the Relative Synonymous Codon Usage (RSCU) for synonymous codons. RNA editing sites in the protein-coding genes (PCGs) of zicaitai and seven other Brassica mitogenomes (*Brassica carinata*, *B. juncea*, *B.* napus, *B. oleracea*, *B. rapa* subsp. *Oleifera*, *B. nigra*, and *B. rapa* subsp. *Nipposinica*) were predicted using the online resource http://prep.unl.edu/ (Mower, 2005).

### 2.5 Ka/Ks analysis and phylogenetic tree construction

The nonsynonymous (Ka) and synonymous (Ks) substitution rates for each PCG beteen zicaitai and seven other reported Brassica species (*B. rapa subsp. nipposinica, B. rapa subsp. oleifera, B. juncea, B. napus, B. oleracea, B. carinata* and *B. nigra*) were calculated using DnaSP (Librado, 2009). A total of 21 complete mitogenomes (**Supplementary Table S5**) were utilized to determine the phylogenetic position of zicaitai. The maximum likelihood evolutionary tree was constructed using 30 conserved mitochondrial PCG genes (*atp1*, *atp4*, *atp6*, *atp8*, *atp9*, *ccmB*, *ccmC*, *ccmFc*, *ccmFn*, *cob*, *cox1*, *cox2*, *cox3*, *matR*, *nad1*, *nad2*, *nad3*, *nad4*, *nad4L*, *nad5*, *nad6*, *nad7*, *nad9*, *rpl2*, *rpl5*, *rps12*, *rps14*(-), *rps3*, *rps4*, and *rps7*) present across the 21 analyzed species. The mafft software (version 7.427, –auto mode) was employed for multiple sequence alignment of inter-species sequences. The aligned sequences were concatenated, and trimAl (version 1.4, -0.7 gt) was used for refinement. The jmodeltest-2.1.10 software was then utilized to select the best-fit model, which was identified as the GTR model. Finally, the RAxML v8.2.10 software (https://cme.h-its.org/exelixis/software.html) was used with the GTRGAMMA model and a bootstrap value of 1000 to construct the maximum likelihood evolutionary tree.

### 2.6 Genome alignments

The zicaitai mitogenome was compared against the chloroplast genome of zicaitai (OP729397.1) using BLASTN 2.9.0+ with the following criteria: a matching rate of ≥70%, an E-value of ≤1e-5, and a sequence length of ≥40 base pairs (Wick et al., 2017). To identify potential regions of nuclear origin within the zicaitai mitogenome, a BLASTN search (with a maximum E-value of 1e-50) was also conducted against all contigs from the previously sequenced zicaitai nuclear genome. Sequences longer than 250 base pairs with a pairwise similarity of >80% were subjected to annotation in the GenBank database to identify their potential nuclear origins.

## 3 Results

### 3.1 Genomic features of the zicaitai mitogenome

**Figure 1:**
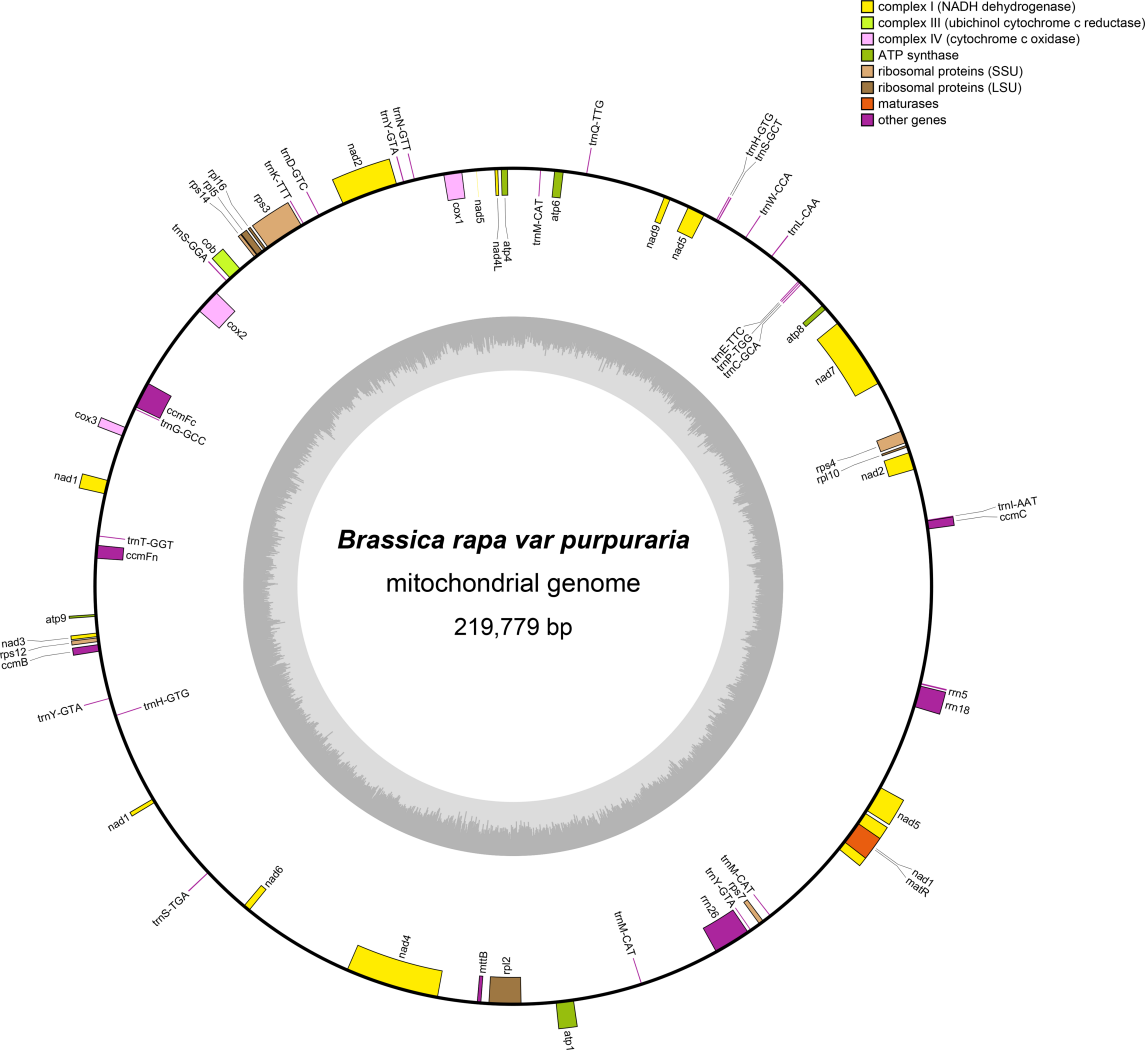
Circular map of the zicaitai mitogenome. Genes shown on the outside and inside of the circle are transcribed clockwise and counterclockwise, respectively. The dark gray region in the inner circle depicts GC content.

The mitogenome of zicaitai, as determined through sequencing with both the Oxford Nanopore and Illumina platforms, has been deposited in the GenBank database under the accession number OP729396.1. This complete mitogenome spans a length of 219,779 base pairs (bp) and exhibits the characteristic circular conformation, as depicted in **Figure 1**. The mitochondrial DNA (mtDNA) nucleotide composition is comprised of adenine (A) at 27.45%, guanine (G) at 22.32%, cytosine (C) at 22.92%, and thymine (T) at 27.31%, with a GC content of 45.24% (**Table 1**). Protein-coding genes (PCGs), introns, tRNA genes, rRNA genes and non-coding regions account for 13.22%, 12.86%, 0.79%, 2.34% and 70.79% of the whole mitogenome, respectively.

**Table 1:** Nucleobase constitution of the zicaitai mitogenome.

Category of sequence	Base composition (%)					Size in bp (proportion in percentage)
	A	G	C	T	GC	
Whole genome	27.45	22.32	22.92	27.31	45.24	219,779 (100%)
Protein-coding genes	26.34	21.7	20.92	31.03	42.63	29,055 (13.22%)
Introns	25.61	26.86	24.22	23.31	51.08	28,253 (12.86%)
tRNA genes	22.57	28.76	22.86	25.81	51.62	1,728 (0.79%)
rRNA genes	26.63	28.79	22.59	21.99	51.38	5,144 (2.34%)
Non-coding regions	27.9	21.97	22.51	27.62	44.48	155,599 (70.79%)

The zicaitai mitogenome contains 977 open reading frames (ORFs) and 59 genes, including 33 protein-coding genes, 23 tRNA genes, and 3 rRNA genes (**Table 2**). The protein-coding genes are categorized into 9 functional groups: 5 for ATP synthases, 4 for cytochrome c biogenesis, 1 for ubiquinol cytochrome c reductase, 3 for cytochrome c oxidase, 1 for maturase, 1 for transport membrane protein, 9 for NADH dehydrogenase, 4 for LSU ribosomal proteins, and 5 for SSU ribosomal proteins. Additionally, three tRNA genes (trnH-GTG, trnM-CAT, trnY-GTA) occur in two or three copies and are located within repeat sequences (**Figure 1**).

**Table 2:** Gene profile and organization of the zicaitai mitogenome.

Gene category	Gene name	Length	Start codon	Stop codon	Amino acid
ATP synthase	*atp1*	1524	ATG	TAG	508
	*atp4*	579	ATG	TAA	193
	*atp6*	786	ATG	TAA	262
	*atp8*	477	ATG	TGA	159
	*atp9*	225	ATG	TGA	75
Cytochrome c biogenesis	*ccmB*	621	ATG	TAA	207
	*ccmC*	744	ATG	TGA	248
	*ccmFc*	1329	ATG	CGA(TGA)	443
	*ccmFn*	1146	ATG	TGA	382
Ubiquinol cytochrome c reductase	*cob*	1182	ATG	TGA	394
Cytochrome c oxidase	*cox1*	1584	ATG	TAA	528
	*cox2*	783	ATG	TAA	261
	*cox3*	798	ATG	TGA	266
Maturases	*matR*	1974	ATG	TAG	658
Transport membrane protein	*mttB*	360	ATG	TAG	120
NADH dehydrogenase	*nad1*	978	ACG(ATG)	TAA	326
	*nad2*	1467	ATG	TAA	489
	*nad3*	357	ATG	TAA	119
	*nad4*	1488	ATG	TGA	496
	*nad4L*	303	ATG	TAA	101
	*nad5*	2010	ATG	TAA	670
	*nad6*	618	ATG	TAA	206
	*nad7*	1185	ATG	TAG	395
	*nad9*	573	ATG	TAA	191
Ribosomal proteins (LSU)	*rpl10*	225	ATG	TAG	75
	*rpl16*	249	ATG	TAA	83
	*rpl2*	1050	ATG	TGA	350
	*rpl5*	558	ATG	TAA	186
Ribosomal proteins (SSU)	*rps12*	378	ATG	TGA	126
	*rps14*	303	ATG	TAG	101
	*rps3*	1665	ATG	TAG	555
	*rps4*	1089	ATG	TAA	363
	*rps7*	447	ATG	TAA	149
Ribosomal RNAs	*rrn18*	1847	–	–	–
	*rrn26*	3176	–	–	–
	*rrn5*	121	–	–	–
Transfer RNAs	*trnC-GCA*	71	–	–	–
	*trnD-GTC*	74	–	–	–
	*trnE-TTC*	72	–	–	–
	*trnG-GCC*	72	–	–	–
	*trnH-GTG (2)*	74/74	–	–	–
	*trnI-AAT*	69	–	–	–
	*trnK-TTT*	73	–	–	–
	*trnL-CAA*	85	–	–	–
	*trnM-CAT (3)*	73/74/74	–	–	–
	*trnN-GTT*	72	–	–	–
	*trnP-TGG*	75	–	–	–
	*trnQ-TTG*	72	–	–	–
	*trnS-GCT*	88	–	–	–
	*trnS-GGA*	87	–	–	–
	*trnS-TGA*	87	–	–	–
	*trnT-GGT*	69	–	–	–
	*trnW-CCA*	74	–	–	–
	*trnY-GTA (3)*	83/68/68	–	–	–

Numbers after gene names are the number of copies.

Mitogenomic sequence-based collinearity analysis has uncovered a high degree of collinearity between the mitogenome of zicaitai and those of *B. rapa subsp. nipposinica*, *B. rapa subsp. oleifera*, and notably, *B. juncea*, which is a distinct species from zicaitai, exhibiting minimal genomic rearrangement characteristics. In contrast, substantial rearrangements were observed when comparing zicaitai with other Brassica species (**Supplementary Figure S1**).

### 3.2 Codon usage analysis of PCGs

The zicaitai mitogenome’s protein-coding genes (PCGs) span a total of 29,055 bp. The majority of these PCGs initiate with the standard ATG start codon and terminate with the common stop codons TAA, TGA, or TAG. Exceptions include the nad1 gene with an ACG start codon and the *ccmFc* gene with a CGA stop codon; both exhibit the phenomenon of C to U RNA editing (**Table 2**).

**Figure 2:**
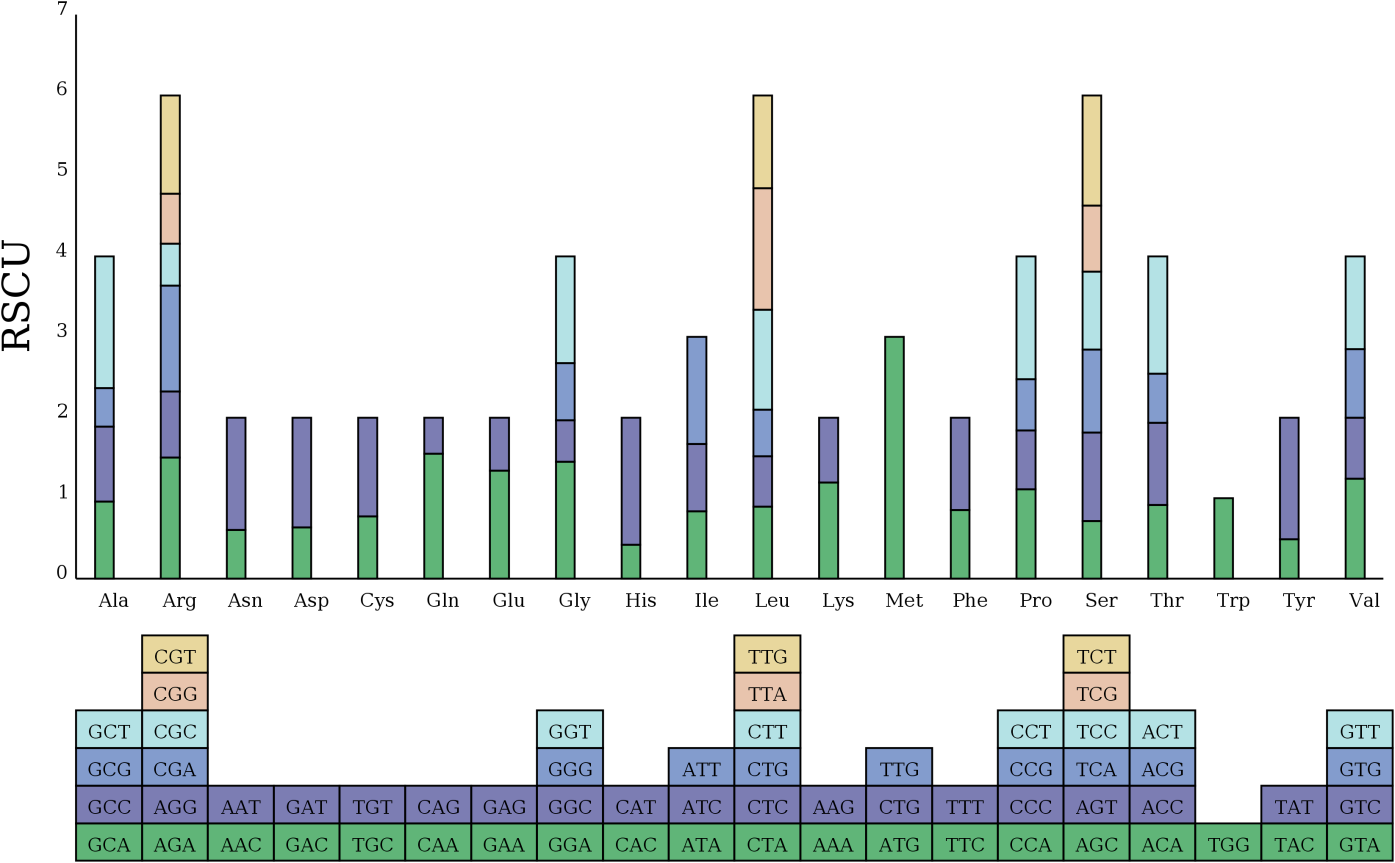
Relative synonymous codon usage (RSCU) in the zicaitai mitogenome. Codon families are shown on the x-axis. RSCU values represent the observed frequency of a specific codon in relation to the expected frequency if synonymous codons were used uniformly.

Additionally, we examined the relative synonymous codon usage (RSCU) for the 33 PCGs in the zicaitai mitogenome (**Figure 2**). Excluding termination codons, these 33 PCGs encode for 9,652 codons. The analysis indicated that leucine (Leu) (10.91%), serine (Ser) (8.87%), and arginase (Arg) (6.48%) are the most frequently used amino acids, as shown in **Supplementary Table S1**. The RSCU values for nearly all NNT and NNA codons exceeded 1.0, with the exceptions of Ala (GCA, 0.96), Thr (ACA, 0.91), Leu (CUA, 0.89), and Ile (AUA, 0.84). A strong bias toward T (U) or A at the third position of the codons in the zicaitai can be observed, a pattern commonly found in land plant mitogenomes (Ma et al., 2022).

### 3.3 Analysis of synonymous and nonsynonymous substitution ratios

**Figure 3:**
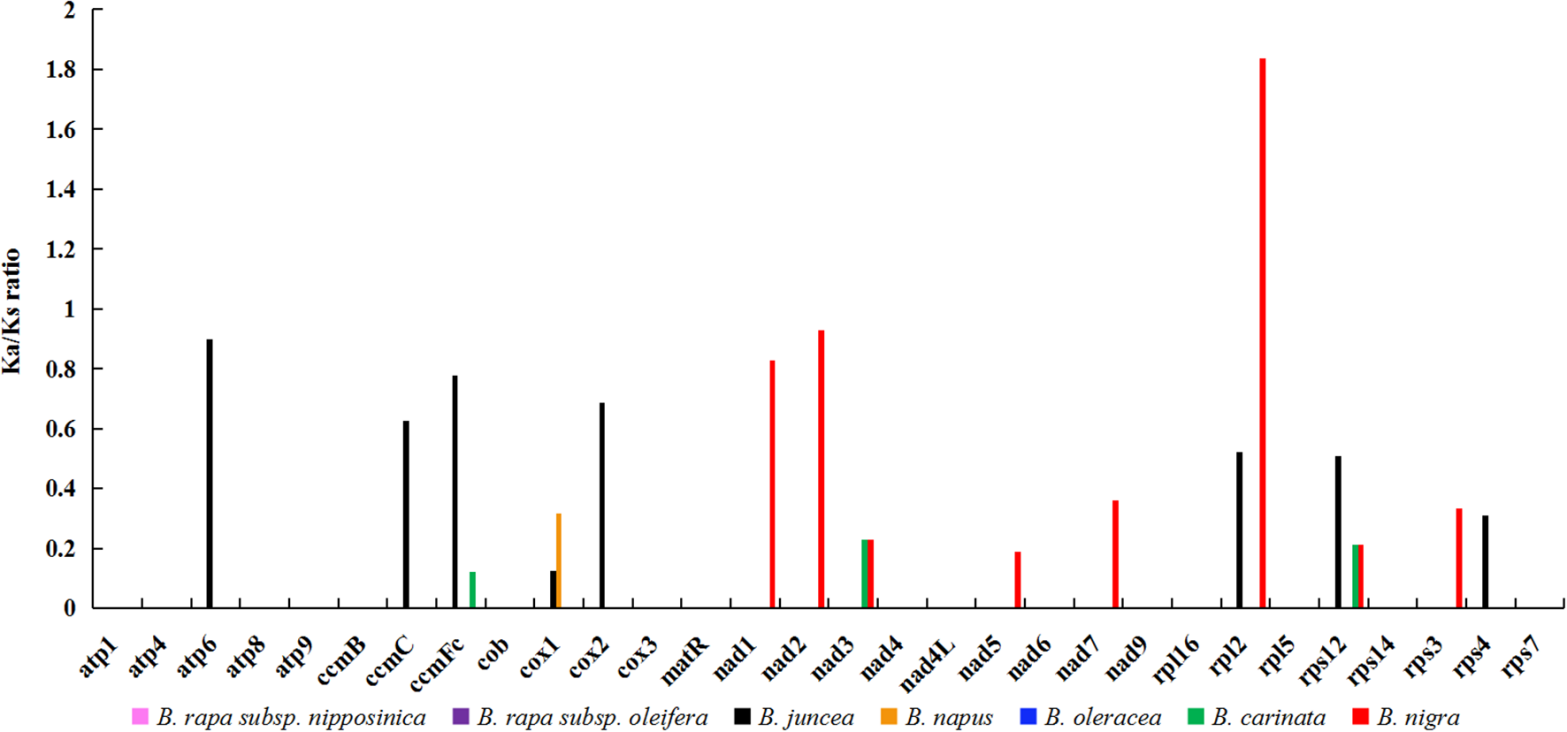
Ka/Ks ratios of 30 protein-coding genes in zicaitai in comparison with *B. rapa subsp. nipposinica*, *B. rapa subsp*. *Oleifera*, *B. juncea*, *B. napus*, *B. oleracea*, *B. carinata* and *B. nigra*, respectively.

In the field of genetics, the ratio of nonsynonymous to synonymous substitutions (Ka/Ks) serves as a valuable tool for elucidating the evolutionary dynamics of genes. In this study, we calculated the Ka/Ks ratio for 30 protein-coding genes (PCGs) shared by zicaitai and seven other Brassica species (*B. rapa subsp. nipposinica, B. rapa subsp. oleifera, B. juncea, B. napus, B. oleracea, B. carinata* and *B. nigra*) (**Figure 3**; **Supplementary Table S2**). The PCGs were found to be highly homologous among zicaitai, *B. rapa subsp. nipposinica*, *B. rapa subsp. oleifera*, and *B. oleracea*, as evidenced by a Ka/Ks ratio of zero for these 30 PCGs. Moreover, the majority of Ka/Ks ratios were below 1.0, implying that these genes have been predominantly under the influence of purifying selection throughout their evolutionary history. In contrast, a single gene, *rpl2*, exhibited a Ka/Ks ratio exceeding 1.0, indicating that it has been subject to positive selection. Lastly, two genes, *atp6* and *nad2*, displayed Ka/Ks ratios approaching 1, suggesting that they have undergone neutral evolution since the time of their common ancestor’s divergence.

### 3.4 Analysis of repeats in the zicaitai

An analysis of long repeats in the zicaitai mitogenome identified 252 dispersed repeats, comprising 108 forward (42.86%) and 144 palindromic (57.14%) repeats; no reverse or complementary repeats were detected. The total length of these long repeats was 16,251 bp, accounting for 7.39% of the length of the complete mitogenome. The average length of the repeats was 64.49 bp, with the longest repeat spanning 2,427 bp. The majority of the repeats (153 repeats, 60.71%) were within the range of 30 to 50 bp in length (**Supplementary Table S3**).

Tandem repeat sequences, also known as satellite DNAs, are formed by the sequential arrangement of short sequences that serve as repeating units, typically ranging from 1 to 200 bases in length. As detailed in **Table 3**, a total of 12 tandem repeats were identified in the zicaitai mitogenome, with lengths varying from 12 to 39 bp, and all of them exhibited a match degree exceeding 95%.

**Table 3:** Tandem repeat sequences in zicaitai mitogenome.

Repeat sequence	Size (bp)	Start site	End site	Percent Matches	Number of copy
AAAGGAGAGGTGCTTTAGCAACTCGACTG	29	28,136	28,225	98	3.1
GCTTTCTTGGTTTGATGAGCTTATACAC	27	49,103	49,176	95	2.7
TGAACTGATAGC	12	61,926	61,950	100	2.1
TCGAGATCTTTGAACCTTTCAG	22	69,116	69,164	96	2.2
ATGTTAGTGTTCAGTATATC	20	71,738	71,775	100	1.9
CTCGAGGAACGC	12	88,938	88,964	100	2.2
CGGAGGCGGGTAAG	14	116,463	116,492	100	2.1
AGATTTTACAAATGGTCTG	19	118,981	119,019	100	2.1
TATCAATTTCATAAGAGAAGAAAGATCGTTTTTTTAAAT	39	119,161	119,271	100	2.8
ATATATCCATTCTCATA	17	138,234	138,272	95	2.3
TAGAAAACTGGCATC	15	147,084	147,113	100	2.0
AACAGATAACAACAGCATATT	21	205,177	205,233	100	2.7

Microsatellites, also known as simple sequence repeats (SSRs), are DNA fragments ranging from 1 to 6 bp in length and are useful molecular markers for studying genetic diversity and identifying species (Ma et al., 2022). In our research, we identified 55 SSRs in the zicaitai mitogenome, comprising 20 mono-, 11 di-, 5 tri-, 18 tetra-, and 1 pentanucleotide repeat (**Table 4**). Over 89% of these SSRs were mono-, di-, or pentanucleotide types. Detailed analysis showed that the A/T repeat accounted for 32.73% (18/55) of the SSRs, followed by the AG/CT repeat at 12.73% (7/55) and the AATG/ATTC repeat at 9.09% (5/55). This is different from the most abundant SSR types in *B. rapa*’s nuclear genome, where A/T and AT/TA repeats are predominant (Gao et al., 2022).

**Table 4:** Frequencies of identified SSR motifs in zicaitai mitogenome.

Motif type	Number of repeats										Total	Proportion (%)
	3	4	5	6	8	10	11	13	14	16		
Monomer						14	2	1	1	2	20	36.4
Dimer			10	1							11	20.0
Trimer		4			1						5	9.1
Tetramer	17	1									18	32.7
Pentamer	1										1	1.8
Total	18	5	10	1	1	14	2	1	1	2	55	100.0

### 3.5 Prediction of RNA editing sites in PCGs

A total of 378 RNA editing sites were identified in the zicaitai mitogenome, all featuring C-to-U changes (**Table 5**). Of these sites, 30.42% (115/378) were located at the first position and 65.34% (247/378) at the second position of codons, while a small fraction, 4.23% (16/378), were found at both positions. There was significant variation in the number of RNA editing sites across different genes, with the highest counts observed in the NADH dehydrogenase genes (nad4, nad5, nad1, nad2, and nad7) and those involved in cytochrome c biogenesis (ccmB and ccmC). In contrast, no RNA editing sites were detected in the atp8 and cox1 genes of zicaitai (**Supplementary Table S4**).

**Table 5:** Prediction of RNA editing sites.

Type	RNA editing	Number	Percentage
hydrophilic	CAC (H) → TAC (Y)	6	12.70%
	CAT (H) → TAT (Y)	16	
	CGC (R) → TGC (C)	6	
	CGT (R) → TGT (C)	20	
hydrophilic-hydrophobic	ACA (T) → ATA (I)	2	45.77%
	ACC (T) → ATC (I)	1	
	ACG (T) → ATG (M)	6	
	ACT (T) → ATT (I)	4	
	CGG (R) → TGG (W)	21	
	TCA (S) → TTA (L)	53	
	TCC (S) → TTC (F)	16	
	TCG (S) → TTG (L)	38	
	TCT (S) → TTT (F)	32	
hydrophilic-stop	CGA (R) → TGA (X)	1	0.26%
hydrophobic-hydrophilic	CCA (P) → TCA (S)	6	8.73%
	CCC (P) → TCC (S)	5	
	CCG (P) → TCG (S)	5	
	CCT (P) → TCT (S)	17	
hydrophobi	CCA (P) → CTA (L)	34	32.54%
	CCC (P) → CTC (L)	7	
	CCC (P) → TTC (F)	6	
	CCG (P) → CTG (L)	20	
	CCT (P) → CTT (L)	23	
	CCT (P) → TTT (F)	10	
	CTC (L) → TTC (F)	4	
	CTT (L) → TTT (F)	8	
	GCA (A) → GTA (V)	2	
	GCC (A) → GTC (V)	4	
	GCG (A) → GTG (V)	4	
	GCT (A) → GTT (V)	1	
Total	—	378	100%

**Figure 4:**
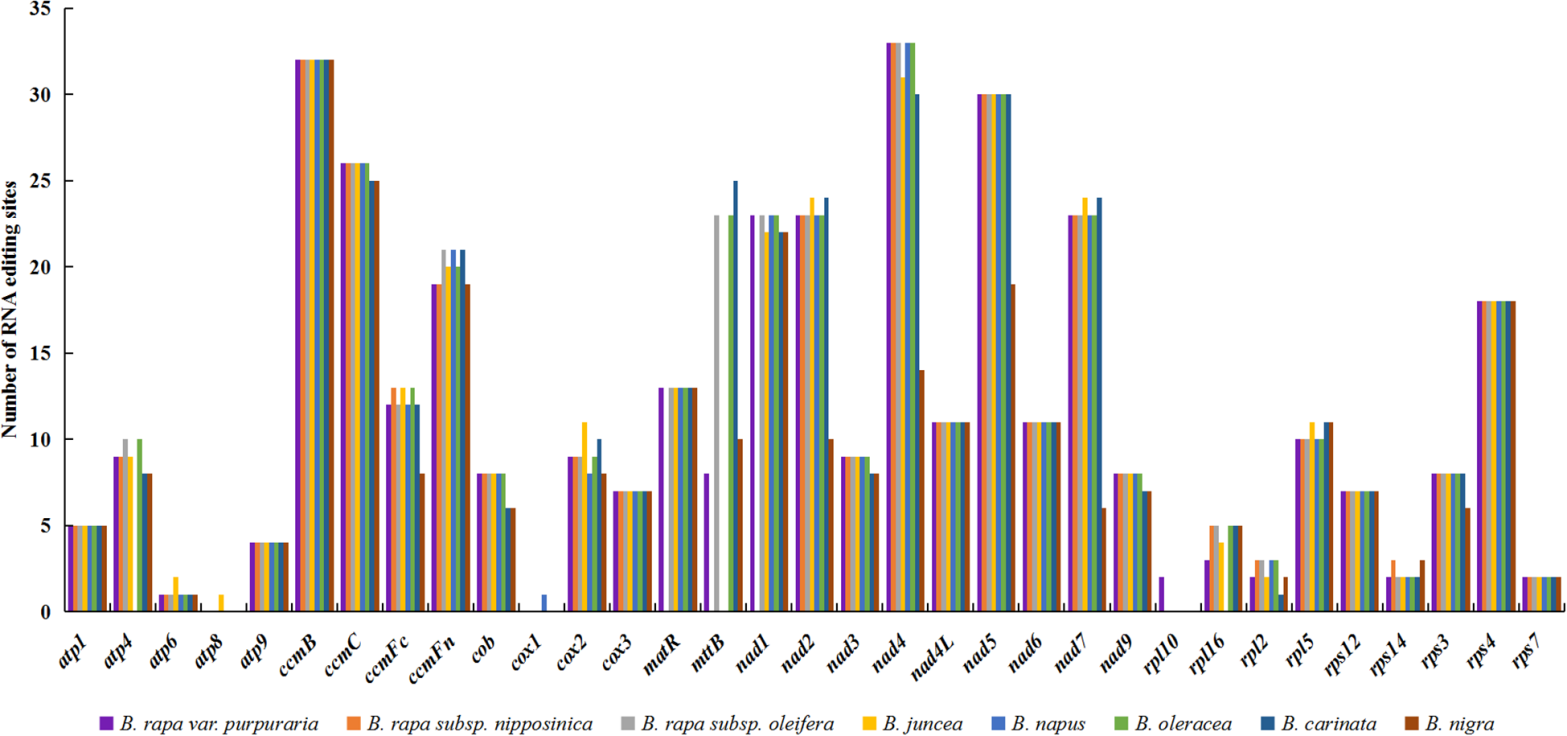
Statistic of RNA editing sites in 33 protein-coding genes present in the mitogenomes of zicaitai and other *Brassica* species.

We examined RNA editing sites across 33 protein-coding genes (PCGs) present in the mitogenomes of zicaitai and seven other *Brassica* species (**Figure 4**), and predicted a total of 2,823 RNA editing sites, with counts varying from 308 in *B. nigra* to 397 in both *B. rapa subsp. Oleifera* and *B. oleracea*. Zicaitai and *B. juncea* had the most similar counts, with 378 and 375 sites respectively.

### 3.6 Phylogenetic analysis

**Figure 5:**
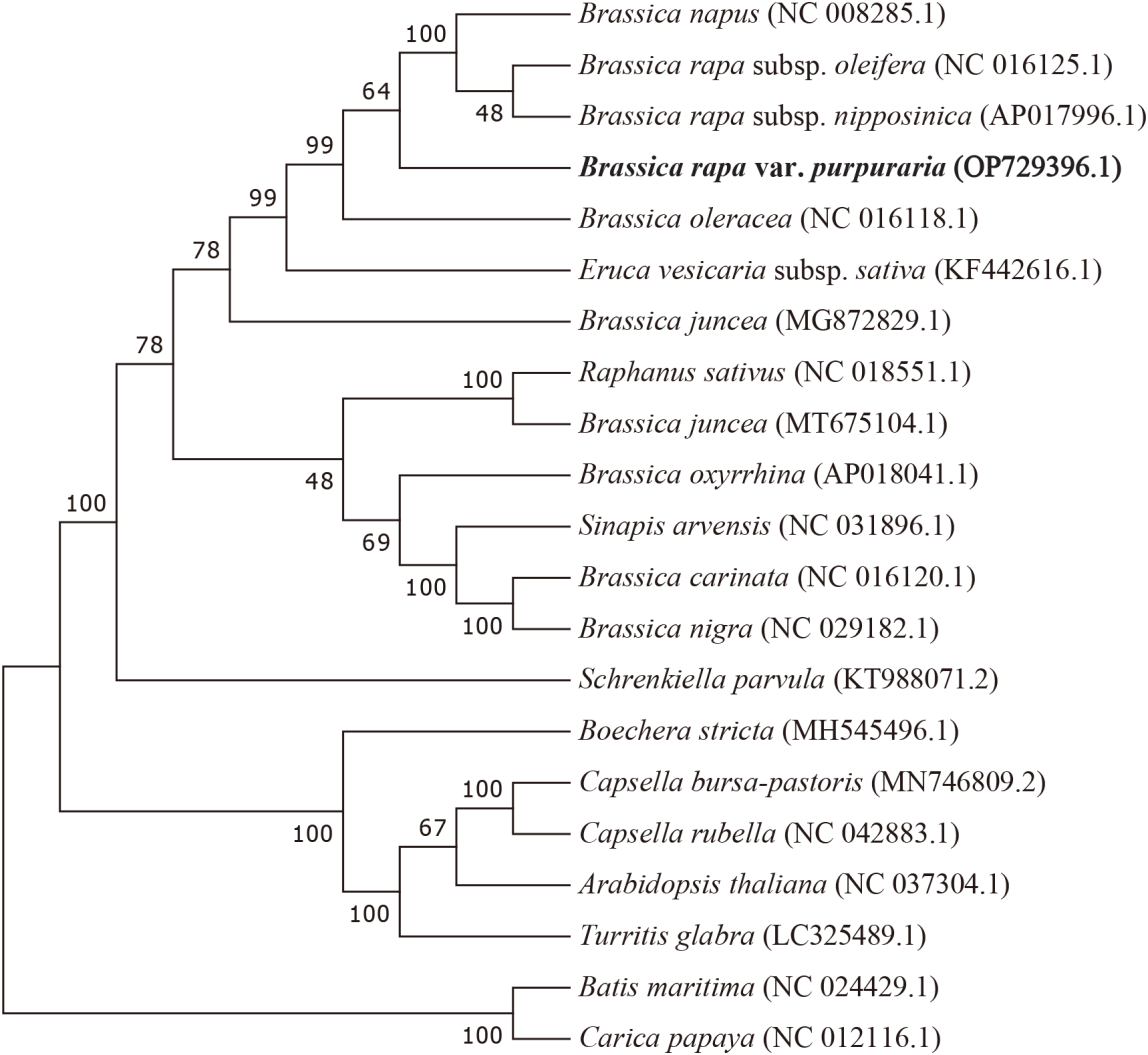
Maximum-likelihood phylogenetic tree based on the sequences of 30 single-copy orthologous genes shared by 21 species in genus *Brassica*. Numbers at nodes are bootstrap support values. The position of zicaitai (*B. rapa* var. *Purpuraria*) is indicated in bold.

To ascertain the phylogenetic position of zicaitai, we retrieved 20 plant mitogenomes from GenBank (https://www.ncbi.nlm.nih.gov/genome/browse/) and constructed a phylogenetic tree using a set of 30 conserved single-copy orthologous genes that are present across all 21 analyzed mitogenomes (**Supplementary Table S5**). As depicted in **Figure 5**, 13 out of 18 nodes within the constructed tree had bootstrap support values exceeding 70%, with 9 nodes achieving full support at 100%. The phylogenetic analysis robustly endorses the close phylogenetic ties between species in genus *Brassica*. Furthermore, it also indicated a close relationship between *B. rapa* and *B. napus*, aligning with the findings of Chang et al. (2011), who suggested that *B. napus* possesses a primary mitotype highly similar to that of *B. rapa*. Collectively, our mitogenomic analysis lays a valuable groundwork for future studies on the phylogenetic relationships within the *Brassica* genus.

### 3.7 Sequence transfer events between the nuclear and organellar genomes in zicaitai

**Figure 6:**
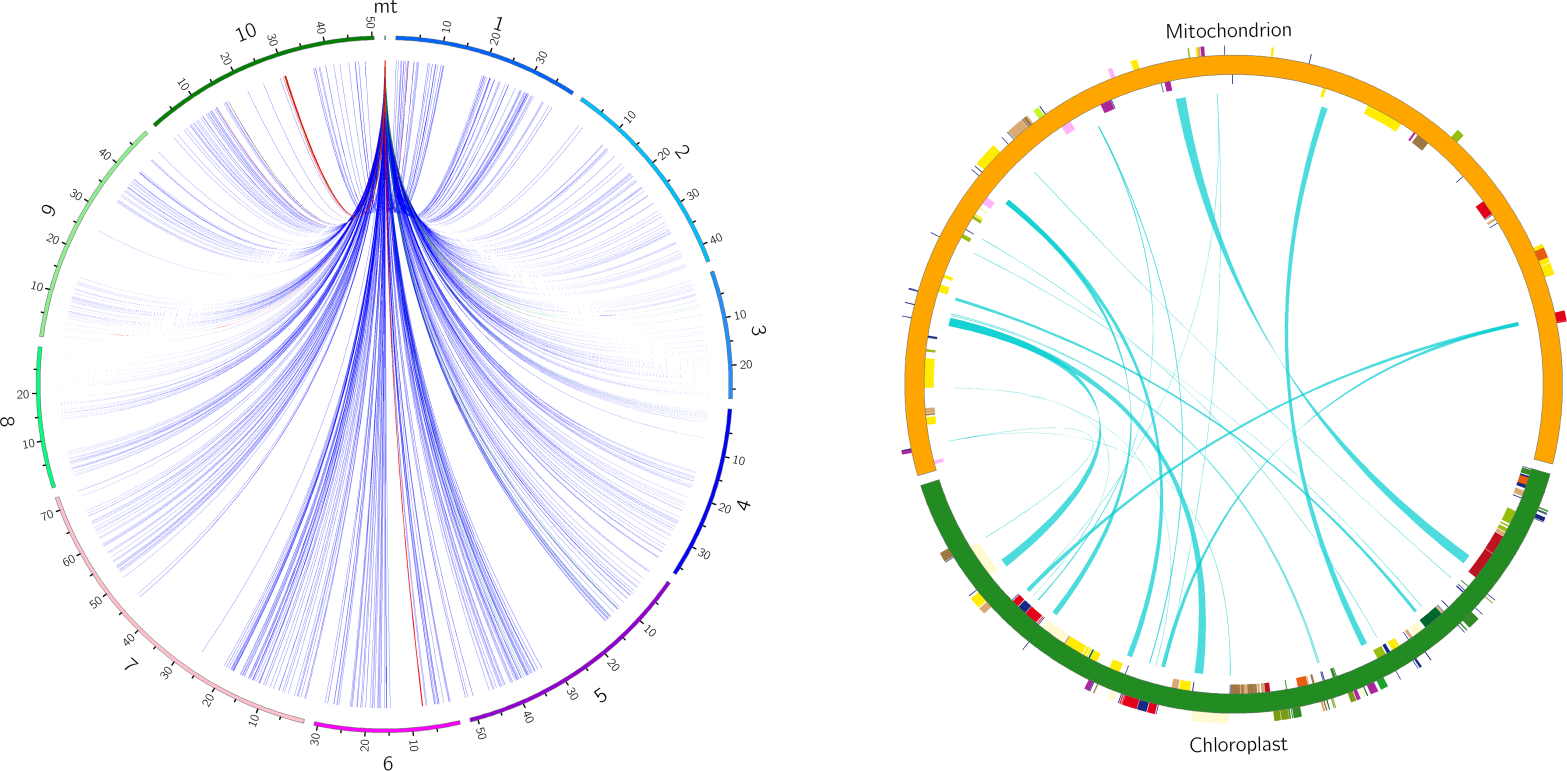
Homologous fragments identified between zicaitai mitogenome and chromosomes (left) and between the mitogenome and chloroplast genome (right). Zicaitai’s ten chromosomes are labeled numerically from 1 to 10 on the left circle. Both circles depict the linkage of homologous fragments across various genome types, connected by curves.

There are six potential directions for gene transfer between nuclear, mitochondrial and chloroplast genomes during plant evolution. To gain a deeper understanding of the sequence transfer events in zicaitai, we conducted a search using the zicaitai mitogenome sequences as queries against the zicaitai nuclear and chloroplast genomes. Our search yielded 1040 hits, encompassing 267,131 base pairs (bp) of sequences shared by both the nuclear genome and the mitogenome (**Supplementary Table S6**). Based on the alignment between the nuclear and mitochondrial genomes, we observed hits across every chromosome of zicaitai (**Figure 6**). However, the total lengths of these hits and their respective coverage percentages varied among the chromosomes. Notably, Chromosome 6 exhibited the longest cumulative length of hits (137,674 bp), significantly surpassing the lengths found on the other chromosomes.

The zicaitai mitogenome sequence, spanning 219,779 bp, is approximately 1.43 times the length of its chloroplast genome (153,621 bp). We identified 22 fragments totaling 13,323 bp, representing 6.06% of the mitogenome, that appear to be the result of gene exchange between the chloroplast genome and the mitogenome of zicaitai (**Table 6**). These fragments encompass seven complete chloroplast genes: *ycf15*, *trnL-CAA*, *trnN-GUU*, *trnW-CCA*, *trnD-GUC*, *trnM-CAU*, and *trnI-CAU*. The remaining fragments consist of partial gene sequences or intergenic spacer regions in the chloroplast genome. Interestingly, our analysis revealed that DNA migration frequently occurred within the inverted repeat region of the zicaitai chloroplast genome.

**Table 6:** Fragments found to be shared by zicaitai’s chloroplast genome and its mitogenome.

No.	Alignment Length	Identity (%)	Mismatch (bp)	Gap opens	CP Start	CP End	Mt Start	Mt End	gene
1	2186	98.81	26	0	23007	25192	108052	110237	*rpoB*
2	1878	97.60	30	5	91634	93500	33491	31618	*ycf2, ycf15, trnL-CAA*
3	1878	97.60	30	5	143266	145132	31618	33491	*trnL-CAA, ycf15, ycf2*
4	1365	96.41	39	3	53575	54934	142593	141234	*rbcL*
5	1159	99.66	4	0	108069	109227	63318	62160	*trnN-GUU, ycf1*
6	1159	99.66	4	0	127539	128697	62160	63318	*ycf1, trnN-GUU*
7	659	96.06	20	3	40170	40826	38183	37529	*psaA*
8	229	96.94	6	1	103753	103980	89674	89446	*rrn23*
9	229	96.94	6	1	132786	133013	89446	89674	*rrn23*
10	878	74.37	170	43	135617	136469	210654	209807	*rrn16*
11	878	74.37	170	43	100297	101149	209807	210654	*rrn16*
12	91	98.90	1	0	38693	38783	48931	49021	*psaB, psaA*
13	143	86.71	11	8	65123	65264	34480	34345	*trnW-CCA*
14	74	97.30	2	0	29508	29581	71852	71779	*trnD-GUC*
15	118	85.59	17	0	65457	65574	34129	34012	*trnP-UGG*
16	77	92.21	6	0	50951	51027	52690	52614	*trnM-CAU*
17	43	100	0	0	100387	100429	117451	117409	*rrn16*
18	43	100	0	0	136337	136379	117409	117451	*rrn16*
19	76	85.53	7	3	85434	85508	5573	5501	*trnI-CAU*
20	76	85.53	7	3	151258	151332	5501	5573	*trnI-CAU*
21	42	97.62	1	0	134348	134389	17596	17555	*trnI-GAU*
22	42	97.62	1	0	102377	102418	17555	17596	*trnI-GAU*

## 4 Discussion

Mitochondria are the energy-producing factories essential for life processes. Plant mitogenomes have more complex structures than those of animals due to size variation, the presence of repeated sequences and other factors (Kozik et al., 2019; Cheng et al., 2021). The availability of rapid and cost-effective genome sequencing technologies has significantly advanced our understanding of mitogenomes. In this study, we characterized for the first time the complete mitogenome of zicaitai, a distinctive *B. rapa* variety. We found that the zicaitai mitogenome has a similar size and GC content to that of other *Brassica* species (Handa, 2003; Chang et al., 2011) and exhibits high degree of collinearity with the mitogenomes of *B. rapa subsp. nipposinica*, *B. rapa subsp. oleifera*, and a distinct species, *B. juncea*, distinguishing zicaitai from other *Brassica* species (**Supplementary Figure S1**). Similar to most other mitogenomes, most sequences in the zicaitai mitogenome are non-coding. The zicaitai mitogenome, like many others, predominantly consists of non-coding sequences, with protein-coding genes (PCGs) making up 13.22%. This is similar to *B. rapa subsp*. *nipposinica* (12.36%), *B. juncea* (14.85%), and *B. oleracea* (13.44%), yet significantly lower than the 20-23% found in other closely related *Brassica* species/varieties. Intriguingly, zicaitai, although belonging to the same species as *B. rapa subsp. oleifera*, exhibits a lower proportion of protein-encoding sequences compared to the latter (22.99%), suggesting an ongoing process of genetic differentiation within the species, likely driven by domestication.

Our research showed that in the zicaitai mitogenome, all protein-coding genes (PCGs) initiate with the standard ATG start codon, with the sole exception of the *nad*1 gene, which starts with ACG - a common occurrence in angiosperms due to RNA editing (Ye et al., 2017; Bi et al., 2020; Ma et al., 2022). In plants, RNA editing is crucial for gene expression, particularly with the cytidine (C) to uridine (U) transition, which is prevalent in mitochondrial and chloroplast genomes (Ma et al., 2022). We also identified the *cox*1 gene, which is known to involve in horizontal gene transfer among angiosperms and vary in its copy number across species as well as populations within a species (Zhang C. et al., 2020). Further studies are needed to clarify the role played by the cox1 gene in *Brassica* evolution. A single gene (*rpl2*) was discovered in this study to present a Ka/Ks ratio exceeding 1.0, consistent with the previous research on other mitochondrial genes with similarly elevated ratios (Ye et al., 2017; Bi et al., 2016, 2020). The significance of the *rpl2* gene in gene selection and the evolutionary process of *Brassica* species/varieties warrants further investigation.

Repeats, encompassing tandem, short, and large types, are crucial for identifying markers in population and evolutionary studies and are prevalent in mitogenomes (Bi et al., 2016; Ma et al., 2017; Liu et al., 2021). They play key roles in intermolecular recombination within mitochondrial DNA, leading to structural variations and diverse mitogenome sizes (Alverson et al., 2010; Guo et al., 2016). In this study, we found an abundance of repeats in the zicaitai mitogenome, notably one large repeat of 2,427 bp. This repeat is identical in size to the one found in the *B. oleracea* mitogenome, suggesting a close genetic link between *B. rapa* and *B. oleracea*, as depicted in **Figure 5**. Our discovery of SSRs in zicaitai’s organelle genome provides a valuable complement to Gao et al. (2022)’s research, which identified 173,892 SSRs within the nuclear genome of *B. rapa*.

Despite variations in RNA editing site counts across zicaitai and various *Brassica* species or varieties, genes for NADH dehydrogenase and cytochrome c biogenesis consistently exhibit the highest editing frequencies among these species/varieties (**Figure 6**). This pattern mirrors observations from previous studies on the mitogenomes of other plant species (Ma et al., 2022). Furthermore, all identified RNA editing sites in the zicaitai mitogenomes are situated at the first and/or second codon positions. It has been hypothesized by previous researchers that the absence of editing sites at the third codon position may be attributed to the limitations of the PREP-Mt predictive method, rather than their non-existence (Mower, 2005; Bi et al., 2020). Consequently, further investigation employing experimental techniques is warranted to clarify this issue.

Previous research has identified the predominant gene transfer in angiosperms as from organellar to nuclear genomes, with secondary importance given to transfers between nuclear, plastid, and mitochondrial genomes (Martin et al., 1998; Rice et al., 2013; Zhao et al., 2018, 2019; Grewe et al., 2014). In higher plants, the total length of transferred DNA varies from 50 kb in *Arabidopsis thaliana* to 1.1 Mb in rice (Smith et al., 2011). Our study has identified a cumulative 267 kb of DNA sequences transferred from 10 chromosomes into the zicaitai mitogenome, with variations in total length and coverage among the chromosomes. Chromosome 6 contributes the largest portion, amounting to 138 kb, which is 62.6% of the mitogenome’s total length. In contrast, sequences from the chloroplast genome make up only 6.1% of the zicaitai mitogenome. Future studies should explore the coevolution of this chromosome with the mitogenome and its potential implications.

In this study, we found 11 tRNA and 3 rRNA genes common to both the nuclear and mitochondrial genomes in zicaitai (**Supplementary Table S6**) and six tRNA genes shared by the chloroplast genome and the mitogenome. While nuclear-to-mitogenome transfers of tRNA genes have been documented in other plants (Chang et al., 2013; Ma et al., 2022), and chloroplast-to-mitogenome transfers are typical in angiosperms (Bergthorsson et al., 2003; Cheng et al., 2021; Bi et al., 2016), the more extensive migration of these genes in zicaitai suggests a more fluid genetic exchange between different cellular compartments than previously thought. Future research should delve into the mechanisms and evolutionary implications of this genetic fluidity.

## 5 Conclusions

We for the first time conducted a comprehensive characterization of the zicaitai mitogenome, which spans a length of 219,779 bp and encompasses a total of 59 genes, including 33 protein-coding genes, 23 tRNA genes, and 3rRNA genes. The protein-coding genes (PCGs), cis introns, tRNA genes, and rRNA genes constitute 13.22%, 12.86% 0.79% and 2.34%, of the zicaitai mitogenome, respectively. The Ka/Ks analysis showed that among 30 shared mitochondrial PCGs in zicaitai and other *Brassica* species, most have experienced purifying selection. Only the *rpl2* gene showed signs of positive selection, and two genes, atp6 and nad2, exhibited neutral evolution. Phylogenetic analysis based on these 30 PCGs from zicaitai and 20 related species/varieties indicated that *B. rapa* is most closely related to *B. napus*, with *B. oleracea* being the next closest. Furthermore, we detected the migration of genes between the chloroplast and nuclear genomes and the zicaitai mitogenome. The collective findings in this study offer an in-depth understanding of the zicaitai mitogenome, which is instrumental for advancing evolutionary studies within the species *B. rapa*.

## Data Availability

The original mitochondrial genome presented in the study are publicly available. This data can be found in NCBI (https://www.ncbi.nlm.nih.gov/) under the GenBank: OP729396.1 (https://www.ncbi.nlm.nih.gov/nuccore/OP729396.1). The data are publicly available. The datasets presented in this study can be found in NCBI.

## Supplementary material

Supplementary Figure 1 Collinearity analysis of mitogenomic sequences between zicatai and some related species/varieties. In each box, the horizontal axis represents the assembled zicaitai’s mitogenomic sequence, and the vertical axis represents the that of related species/varieties. The red lines within the boxes indicate forward alignments, while the blue lines indicate reverse complement alignments.
