# Supplementary figures and images for: Assembly and comparative analysis of the first complete mitochondrial genome of zicaitai (Brassica rapa var. Purpuraria): insights into its genetic architecture and evolutionary relationships
Source: Front Plant Sci. 2024 Oct 10;15:1475064. doi: 10.3389/fpls.2024.1475064 (PMC11499134; doi:10.3389/fpls.2024.1475064)

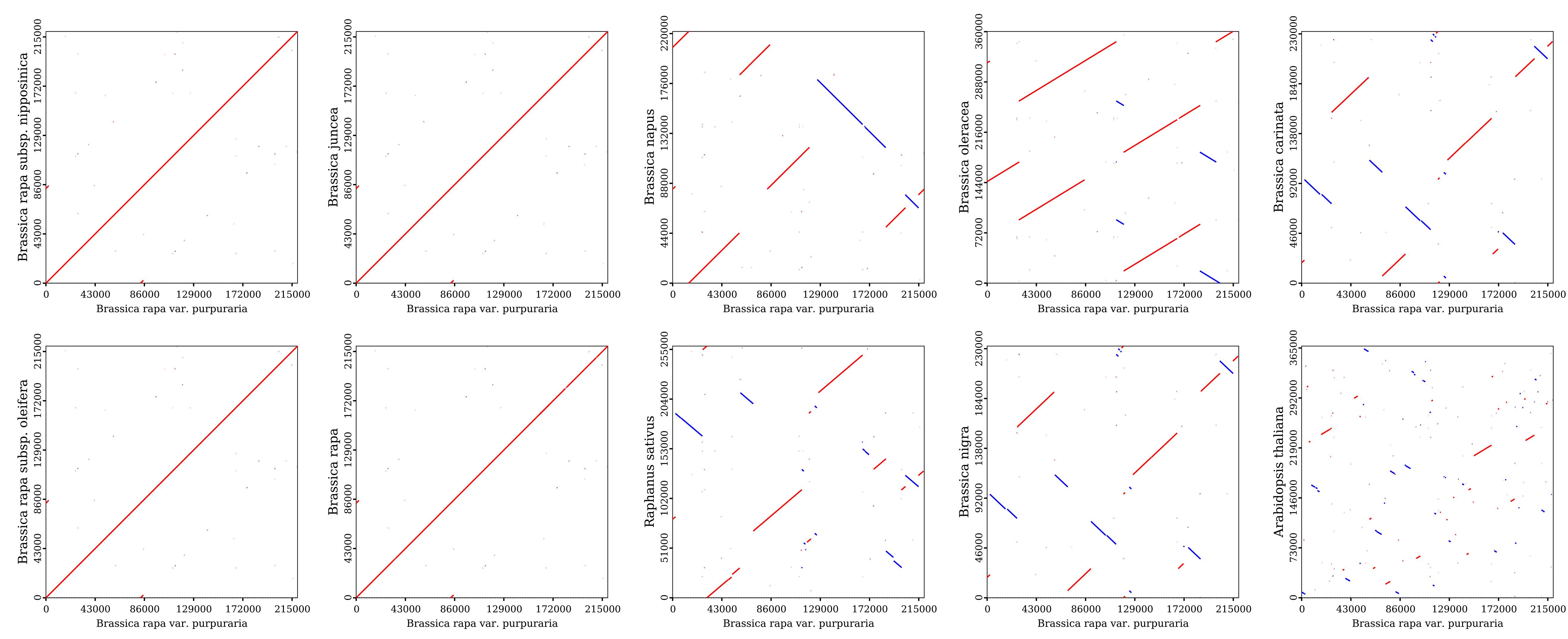

Supplement: Supplementary Figure 1 — Collinearity analysis of mitogenomic sequences between zicatai and some related species/varieties. In each box, the horizontal axis represents the assembled zicaitai’s mitogenomic sequence, and the vertical axis represents the that of related species/varieties. The red lines within the boxes indicate forward alignments, while the blue lines indicate reverse complement alignments. [file Image1.tif]
